# Pentoxifylline as Add-On Treatment to Donepezil in Copper Sulphate-Induced Alzheimer’s Disease-Like Neurodegeneration in Rats

**DOI:** 10.1007/s12640-023-00672-1

**Published:** 2023-10-12

**Authors:** Mohamed M. Elseweidy, Mohamed Mahrous, Sousou I. Ali, Mohamed A. Shaheen, Nahla N. Younis

**Affiliations:** 1 Department of Biochemistry, Faculty of Pharmacy, Zagazig University, Zagazig, 44519 Egypt; 2 Department of Biochemistry, Faculty of Pharmacy, Port-Said University, Port-Said, 42526 Egypt; 3 Department of Histology and Cell Biology, Faculty of Human Medicine, Zagazig University, Zagazig, 44519 Egypt

**Keywords:** Alzheimer’s disease, Donepezil, Pentoxifylline, Copper sulphate, Apoptosis, Neurodegeneration

## Abstract

Alzheimer’s disease (AD), the most common neurodegenerative disorder, is characterized by behavioral, cognitive, and progressive memory impairments. Extensive neuronal loss, extracellular accumulation of insoluble senile amyloid-β (Aβ) plaques, and intracellular neurofibrillary tangles (NFTs) are the major pathological features. The present study aimed to investigate the therapeutic effect of donepezil (DON) and pentoxifylline (PTX) in combination to combat the neurodegenerative disorders (experimental AD) induced by CuSO_4_ intake in experimental rats. Thirty adult male Wistar rats (140–160 g) were used in this study. AD was first induced in rats by CuSO_4_ supplement to drinking water (10 mg/L) for 14 weeks. The AD group received no further treatment. Oral treatment with DON (10 mg/kg/day), PTX (100 mg/kg/day), or DON + PTX for the other three groups was started from the 10th week of CuSO_4_ intake for 4 weeks. Cortex markers like acetylcholine (ACh), acetylcholinesterase (AChE), total antioxidant capacity (TAC), and malondialdehyde (MDA) and hippocampus markers like β-amyloid precursor protein cleaving enzyme 1 (BACE1), phosphorylated Tau (p-tau), Clusterin (CLU), tumor necrosis factor-α (TNF-α), caspase-9 (CAS-9), Bax, and Bcl-2 were measured. The histopathology studies were done by using hematoxylin and eosin and Congo red stains as well as immunohistochemistry for neurofilament. CuSO_4_ induced adverse histological and biochemical changes. The histological injury in the hippocampus was inhibited following the administration of the DON and PTX. The brain tissue levels of AChE, MDA, BACE1, p-tau, CLU, CAS-9, Bax, and TNF-α were significantly increased, while brain tissue levels of ACh, TAC, and Bcl-2 were significantly decreased in CuSO_4_-treated rats as compared with the untreated control group. The effects induced by either DON or PTX on most studied parameters were comparable. Combined treatment of DON and PTX induced remarkable results compared with their individual use. However, more clinical and preclinical studies are still required to further confirm and prove the long-term efficacy of such combination.

## Introduction

Alzheimer’s disease (AD) is the most common form of dementia and degenerative brain illness (Malik et al. 2022). More than 55 million people worldwide suffer from AD and associated dementias with nearly 10 million new cases every year. Over 60% of cases are living in low- and middle-income countries (World Health Organization 2017). As a progressive cognitive dysfunction disease, AD provokes various degrees of damage to patients’ language, visual space, and memory function. These together may result in lowering overall cognitive ability, personality disorders, and significant declines in work, social, and daily life abilities (Moore et al. 2014).

AD has a complex and multifactorial pathophysiology (Gavrilova and Alvarez 2021), where the accumulation of amyloid-β cerebral plaques (Aβ) and neurofibrillary tangles (NFTs) of abnormally insoluble Tau (an axonal protein) are common pathologic features (Knorz and Quante 2022). The engagement of cholinergic neuron in AD pathology resulted in a decline in synaptic acetylcholine (ACh) levels. Depletion of other neurotransmitters, loss of neuronal connections, mitochondrial failure, oxidative stress, inflammation, ischemia, impaired insulin signaling, and cholesterol metabolism abnormalities are all possible contributors to the pathogenesis of AD (Winslow et al. 2011). In other words, multiple pathogenic processes combine their harmful effects to induce neuron destruction in AD; therefore, a successful medication would ideally suppress several processes (Tatulian 2022). Accordingly, a single effective medication against all multiple pathologies may not be available, and a combination of treatments fulfilling this aim may be a better strategy (Weinstein 2017).

Although there are no accessible disease-modifying medications for AD, the currently approved drugs appear to be beneficial in measures of cognition, behavior, and everyday function (Herrmann et al. 2011). These include three cholinesterase inhibitors (galantamine, rivastigmine, and donepezil (DON)) and one N-methyl-d-aspartate receptor antagonist (memantine) (Campos et al. 2016). DON as a powerful and selective acetylcholinesterase (AChE) inhibitor has been demonstrated to be efficient in moving forward cognitive performance in patients with AD (Kwon et al. 2014). In traumatic brain injury, DON reduced the volume of cerebral infarction, protected against neuronal cell death and cognitive shortages, and enhanced adult hippocampal neurogenesis by increasing the level of cAMP-response element-binding protein (CREB) phosphorylation (Kwon et al. 2014).

Drugs that maintain synapse function in the presence of Aβ may provide a therapeutic benefit for patients when used as an adjunct to conventional Aβ lowering treatments (Bate and Williams 2015). Pentoxifylline (PTX), a methylxanthine derivative, is a nonselective phosphodiesterase inhibitor which may cross the blood–brain barrier quickly and effectively after dosage (Alzoubi et al. 2013). PTX has a favorable effect; it inhibits the conversion of cAMP to AMP, increases cAMP levels, and enhances cell function and hemorheology. PTX also increases oxygen transport to ischemic regions by raising intracellular cAMP in red blood cells. Under hypoxia, glycolysis is the primary metabolic process for energy delivery. PTX has been shown to boost glycolysis and respiratory rates, as well as ATP generation and microcirculation (Yao et al. 2016). Multiple cytokine pathways, including those involved in tumor necrosis factor (TNF) and transforming growth factor (TGF) signaling, are disrupted by PTX (Albersen et al. 2011). The current treatment for AD employs PTX to enhance cerebral blood circulation, increase brain cell metabolism, and slow disease development (Bath and Wardlaw 2015). PTX has been found to alleviate cognitive problems due to cerebral ischemia by enhancing blood flow, inducing an anti-inflammatory effect, and minimizing cell death (Yao et al. 2016; Akbari et al. 2020).

Copper (Cu) is a vital element in mammalian nutrition, where a trace amount is necessary for cellular function and survival. It is essential for proper infant growth, brain development, and body immunity. Although Cu has so many functions in biological systems, its concentration must be appropriately regulated to avoid toxicity (Arowoogun et al. 2021). Alterations in brain Cu levels have been implicated in the pathogenesis of several neurological disorders including AD, Parkinson’s, and prion diseases (Squitti 2014; Shao et al. 2018). Excess Cu in the biological system has been linked to the generation of reactive oxygen species (ROS) via the Fenton reaction (Huang et al. 2004; Valko et al. 2005) and is associated with brain oxidative stress and chronic inflammation and hence neuronal damage (Barnham and Bush 2014; Parthasarathy et al. 2014). The oxidized form of copper ions (Cu^2+^) can bind to β-amyloid peptides with high affinity and increase the proportions of β sheet and α-helix structures in amyloid peptides, which can be responsible for β amyloid aggregation. Similarly, Cu can also bind to Tau proteins and promote the formation of NFTs (Bacchella et al. 2020).

The modulating effects of PTX on AD pathogenesis are not known. Moreover, drug combination strategy targeting multiple AD pathogenesis using DON and PTX is not studied yet. The present study, therefore, aimed to (i) compare the effect of PTX and DON that is routinely used to treat AD and (ii) demonstrate the therapeutic potential of PTX and DON combination on multiple brain markers using an experimental AD rat model induced by CuSO_4_ intake.

## Materials and Methods

### Drugs and Chemicals

Donepezil hydrochloride (Pfizer Egypt, Cairo, A.R.E.), pentoxifylline (Trental^®^; Hoechst Orient S.A.E, Cairo, Egypt), and copper sulphate (CuSO_4_; Sigma Aldrich, St. Louis, MO, USA.) were used in this study. All drugs were dissolved in saline and were freshly prepared immediately before use.

### Animals and Experimental Design

Male Wister rats (weighing 140–160 g) were purchased from the animal unit at the Faculty of Veterinary Medicine, Zagazig University, Egypt. Rats were housed (6 per cage) in wire-floored cages at a regulated environment (temperature, 22 ± 2 °C; humidity, 50 ± 5%; night/day cycle, 12 h) with free access to standard pellet diet and tap water ad libitum. All experiments and animal procedures followed the National Institutes of Health guide for the care and use of Laboratory animals (NIH Publications No. 8023, revised 1978) and obtained an ethical approval from the Institutional Animal Care and Use Committee of Zagazig University (ZU-IACUC/3/F/43/2019).

**Fig. 1:**
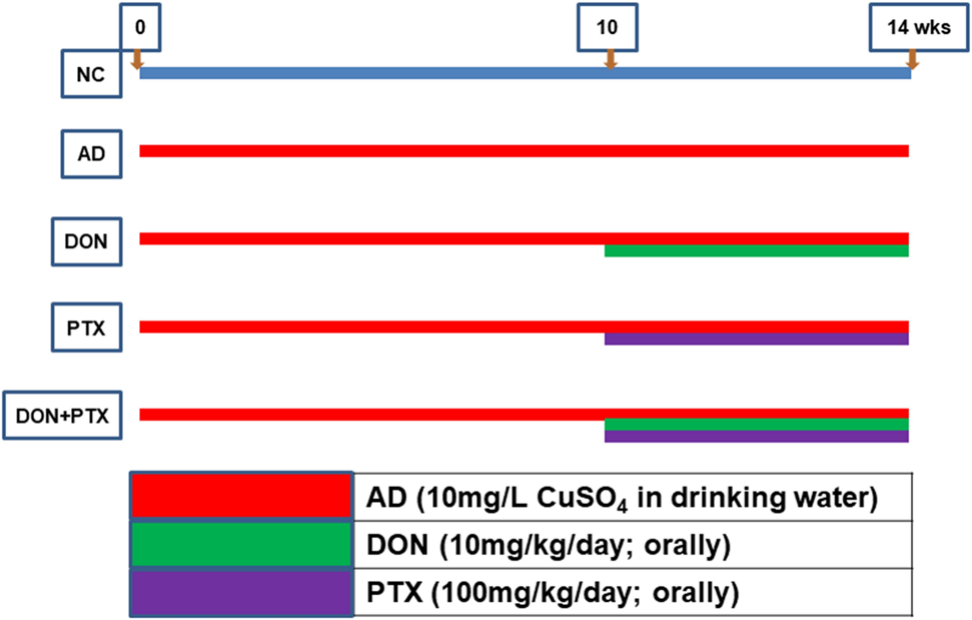
Schematic illustration of experimental groups

Thirty rats were randomly divided into five groups after a 2-week acclimatization period (6 rats per group). The normal control group (NC) received normal drinking water for 14 weeks. The remaining four groups received CuSO_4_ in drinking water (10 mg/L) for 14 weeks to develop AD. One of these groups received saline (vehicle) orally for 4 weeks starting on the 10th week of CuSO_4_ administration and served as the AD group. The remaining three AD groups received drug treatment for 4 weeks orally starting on the 10th week of CuSO_4_ administration as follows: DON group, AD rats received DON (10 mg/kg/day) by oral gavage (Kwon et al. 2014); PTX group, AD rats received PTX (100 mg/kg/day) by oral gavage (Albersen et al. 2011); and DON + PTX group, AD rats received both DON and PTX as previously described. A schematic illustration of the experimental groups was presented in Fig. 1.

### Y-maze Behavioral Test

A black Y-shaped plastic device with three arms (50 cm long, 32 cm high, and 16 cm broad) at 120° angles makes up the maze. A Y-maze behavioral test was performed 2 days prior to scarification. Each rat was first placed in the maze’s center and given 5 min to roam around freely. The order in which the arms were entered was recorded (four paws had to be inside the arm for a valid entry). If a rat entered three different arms in a row, it was considered a spontaneous alternation. The percentage of spontaneous alternation (spatial cognition ability) was calculated using the following formula: (Kang et al. 2016) Spatial cognition ability (%) = Actual alteration/(total number of arm entries-2) × 100.

### Sampling

Rats were sacrificed by decapitation 2 days after the Y-maze behavior test (described below) at the end of 14 weeks. The brains were immediately removed and were rinsed in an ice-cold saline solution. The cerebral cortex and hippocampus were carefully dissected, and one part was frozen in liquid nitrogen and stored at − 80 °C for subsequent biochemical assessment; the other part was kept in 10% buffered formalin for histopathology and immunopathology examinations. The cortical and hippocampus tissues were homogenized in ice-cold phosphate buffer pH 7.4 to obtain a 10% homogenate for biochemical investigations.

### Cholinergic Activity

The commercial rat-specific Sandwich-ELISA kits (Elabscience, TX, USA) were used to quantify the levels of ACh (Catalog No: E-EL-0081) and AChE (Catalog No: E-EL-R0355) in the brain cortex. The instructions of the manufacturer were followed.

### Apoptosis and Inflammation

ELISA kits (Elabscience, TX, USA) were used to measure the expression of Bax (Catalog No: E-EL-R0098), Bcl-2 (Catalog No: E-EL-R0096), CAS-9 (Catalog No: E-EL-R0163), and TNF-α (Catalog No: E-EL-R0019) in the brain hippocampus.

### BACE1, CLU, and p-tau

The hippocampus content of AD biomarkers was measured using commercial ELISA kits for rat BACE1 (Catalog No: LS-F15104; Lifespan Biosciences, Inc., WA, USA), rat CLU (Catalog No: ELR-Clusterin; Raybiotech, GA, USA), and rat p-tau (Catalog No: E-EL-R1090; Elabscience, TX, USA).

### Oxidative Stress Markers

The total antioxidant capacity (TAC) and lipid peroxidation, expressed as malondialdehyde (MDA), were measured in brain cortical tissue using commercially available kits purchased from Biodiagnostic Co., Giza, Egypt (Catalog No: TA 2512 and MD 2529, respectively).

### Histopathology Studies

Hippocampus, coronal sections (5 µm thick) were processed using conventional histological procedures and stained with hematoxylin and eosin (H and E) (Serrano-Pozo et al. 2011). Congo red staining was used to reveal β-amyloid deposits in hippocampus tissue (Khurana et al. 2001). The slides were examined using a light microscope (magnification 400 ×). Three random fields were captured from each section (18 different fields for each group) which were analyzed using the ImageJ software to count the number of neuron cells with histological features of apoptosis (apoptotic-like neurons) in hippocampus tissue and the amount of amyloid deposits (Ferreira and Rasband 2012). The apoptotic-like neurons were identified as being smaller than the surrounding cells with a small pyknotic nucleus (darkly stained nucleus due to chromatin condensation) and intensely stained acidophilic cytoplasm (due to cytoplasmic condensation). These were also identified by the poorly demarcated or fragmented nuclei “apoptotic bodies” (Elmore et al. 2016).

### Immunohistochemistry

Hippocampus sections (5 µm thick) were incubated overnight at 4 °C in a humidified chamber with mouse monoclonal light neurofilament primary antibody (Abcam, ab7255). A biotin-labelled goat anti-mouse IgG (Abcam, ab6788) was used as a secondary antibody after washing with phosphate buffer saline (PBS). The sections were examined microscopically for specific staining (Olympus CX40; Olympus, Tokyo, Japan). Using the ImageJ software, the integrated optical density (IOD) value of the collected pictures was measured in 18 random visual fields from each group (three fields/rat).

### Statistical Analysis

GraphPad Prism version 5.0 was used for statistical analyses (GraphPad Software, San Diego, USA). Normality distribution was first checked visually using Q-Q plot and was tested using the Shapiro–Wilk test. The analysis of variance (ANOVA) was used to compare data, followed by Tukey’s post hoc test taking *P* < 0.05 as statistically significant. All results were graphically displayed as mean ± SD.

## Results

### Effects of DON and PTX on Spatial Learning and Memory Ability

**Fig. 2:**
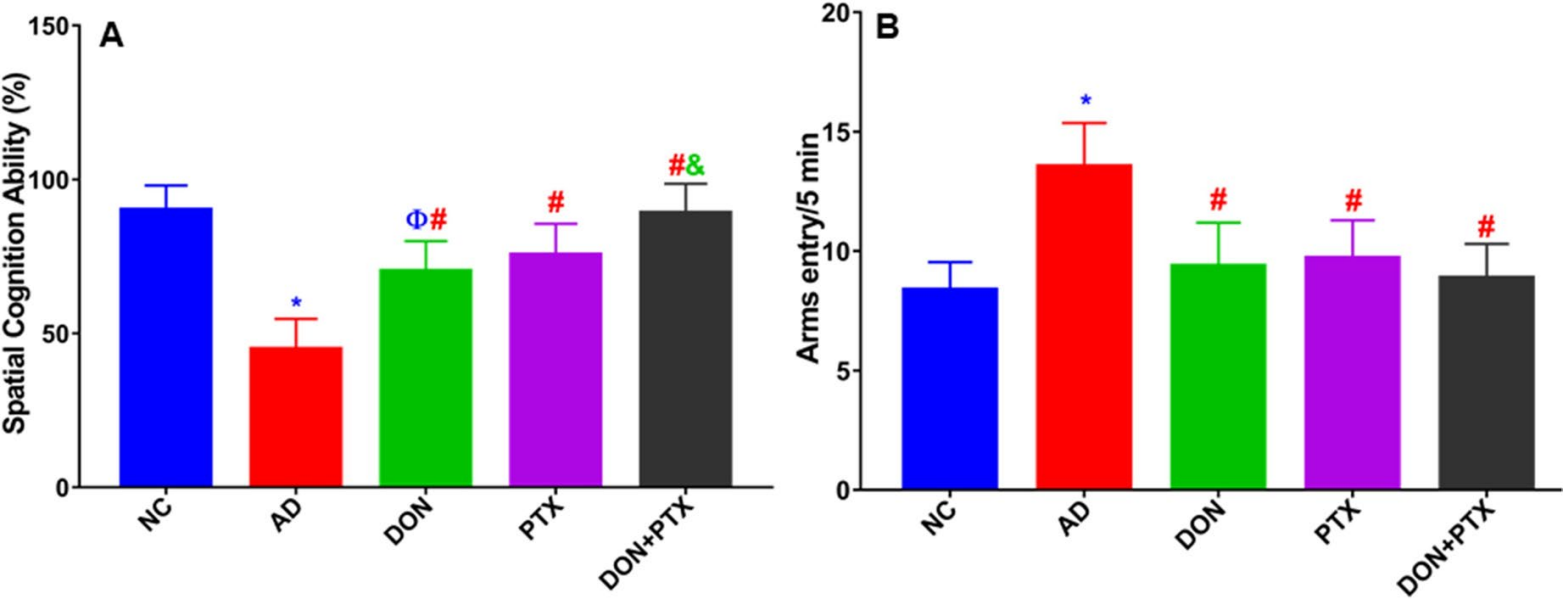
Y-maze test results showing **A** percentage spatial cognition ability and **B** locomotor activity in rats with CuSO_4_-induced AD and treated with DON, PTX, or their combination for 4 weeks. Results are expressed as mean ± SD, *n* = 6. *P < 0.001 and ^Ф^*P* < 0.05 compared to the NC group, ^#^*P* < 0.001 compared to the AD group, ^&^*P* < 0.001 compared to the DON group

The intake of CuSO_4_ for 14 weeks resulted in an impairment of percentage spatial cognition ability and increased the number of arm entries implying an impairment of locomotor activity compared to the normal control group (*P* < 0.001). Treatment with PTX, DON, or their combination significantly improved the percentage of spatial cognition ability and locomotor activity (arm entry) as compared to the AD group (*P* < 0.001). The combined DON and PTX treatment showed better spatial cognition ability than that produced by DON alone (*P* < 0.001) as shown in Fig. 2A and B, respectively.

### Effects of DON and PTX on Cholinergic Activity

**Fig. 3:**
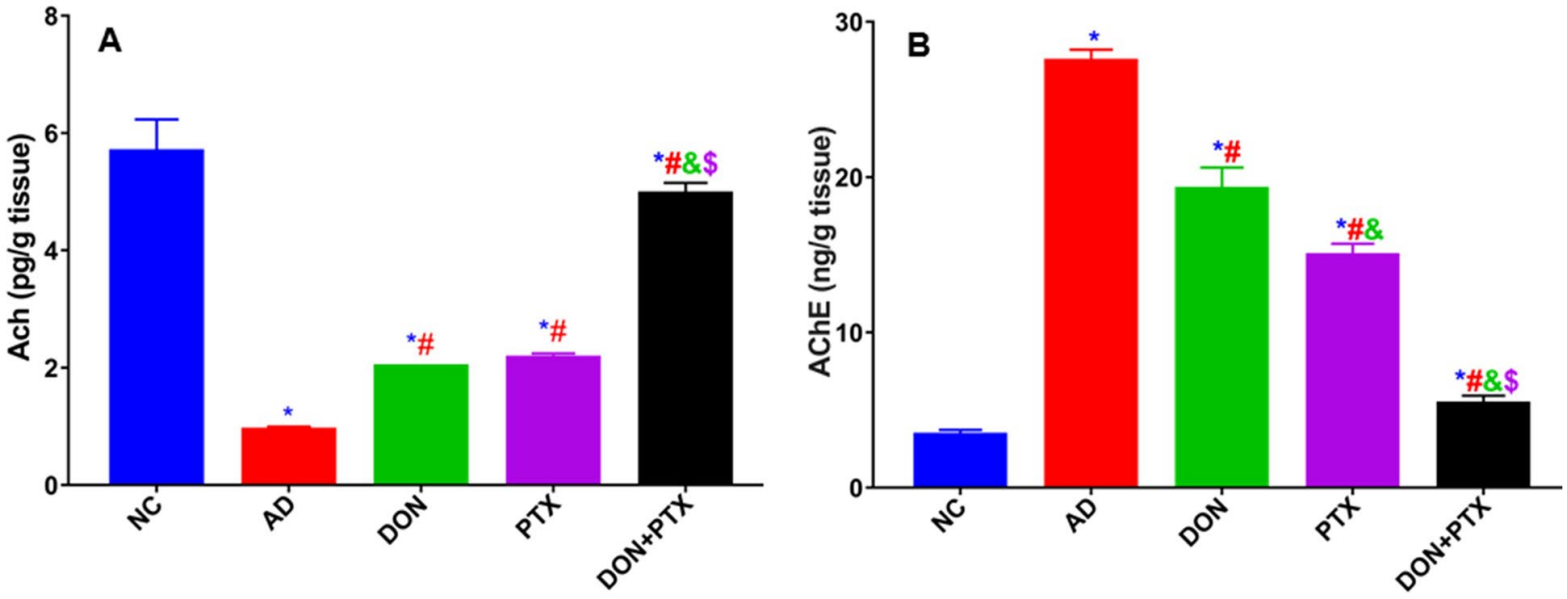
Cholinergic activity **A** Cortical ACh and **B** AChE content in rats received CuSO_4_ (AD group) and treated with DON, PTX, or their combination for 4 weeks. Results are expressed as mean ± SD, *n* = 6. *P < 0.001 compared to the NC group, ^#^*P* < 0.001 compared to the AD group, ^&^*P* < 0.001 compared to the DON group, ^$^*P* < 0.001 compared to the PTX group

Rats received CuSO_4_ (AD rats) showed decreased cortical ACh along with increased AChE content compared to the normal control group (*P* < 0.001). Treatment with PTX, DON, or their combination significantly increased ACh and decreased AChE as compared to the AD group (*P* < 0.001). The combination of DON and PTX demonstrated remarkable results compared with individual treatments (*P* < 0.001) as shown in Fig. 3.

### Effects of DON and PTX on AD Biomarkers

**Fig. 4:**
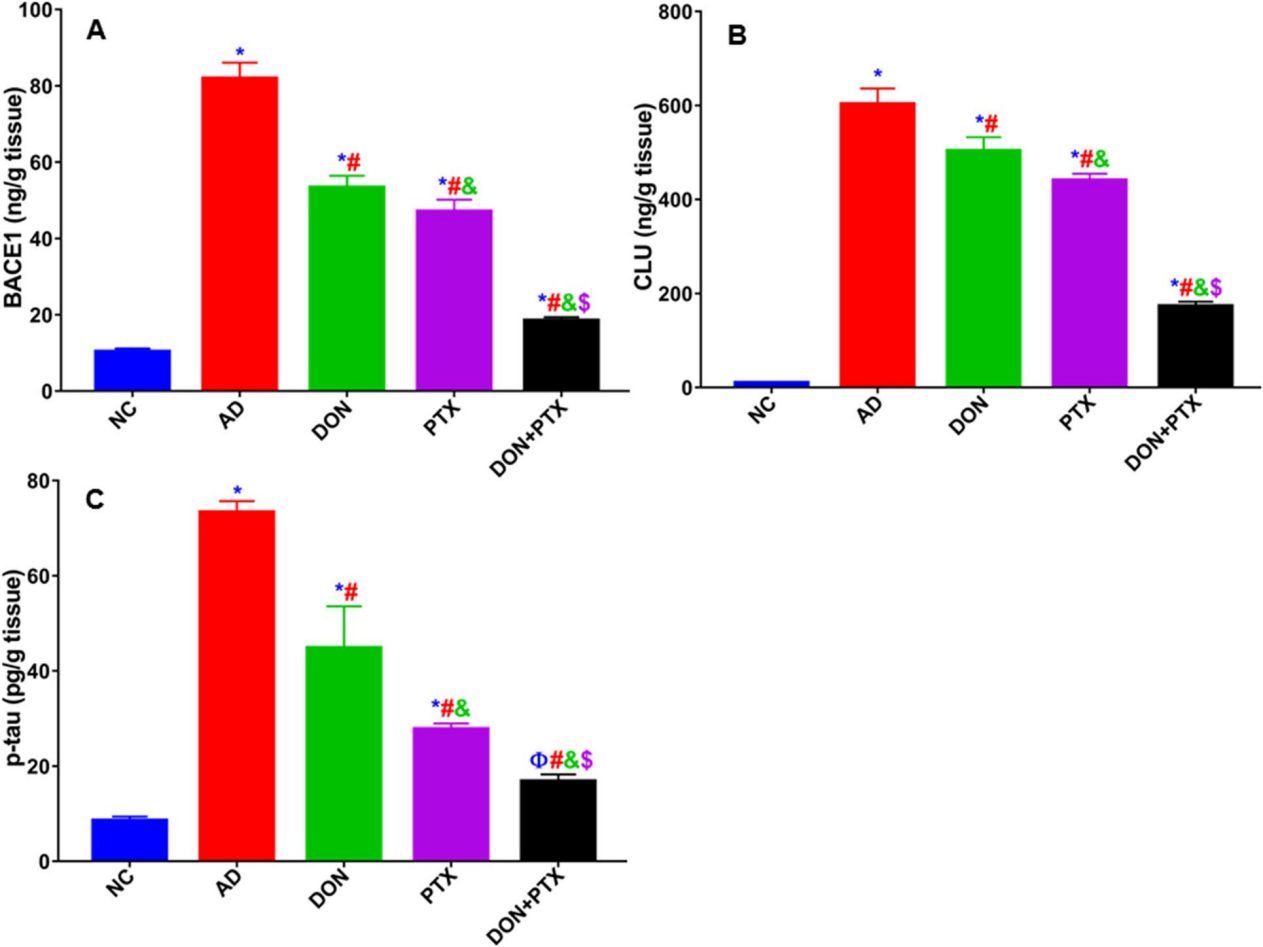
The brain hippocampus contents of **A** BACE1, **B** CLU, and **C** p-tau in rats received CuSO_4_ and treated with DON, PTX, or their combination for 4 weeks. Results are expressed as mean ± SD, *n* = 6. *P < 0.001 and ^Ф^*P* < 0.05 compared to the NC group, ^#^*P* < 0.001 compared to the AD group, ^&^*P* < 0.001 compared to the DON group, ^$^*P* < 0.001 compared to the PTX group

AD rats showed notably increased brain hippocampus contents of BACE1, CLU, and p-tau as compared to NC rats (*P* < 0.001). As compared to the untreated AD group, the individual treatment with DON, PTX, or their combination significantly decreased BACE1, CLU, and p-tau levels (*P* < 0.001). Combination treatment (DON + PTX) demonstrated remarkable (*P* < 0.001) results compared with individual ones (Fig. 4).

### Effects of DON and PTX on TNF-α

**Fig. 5:**
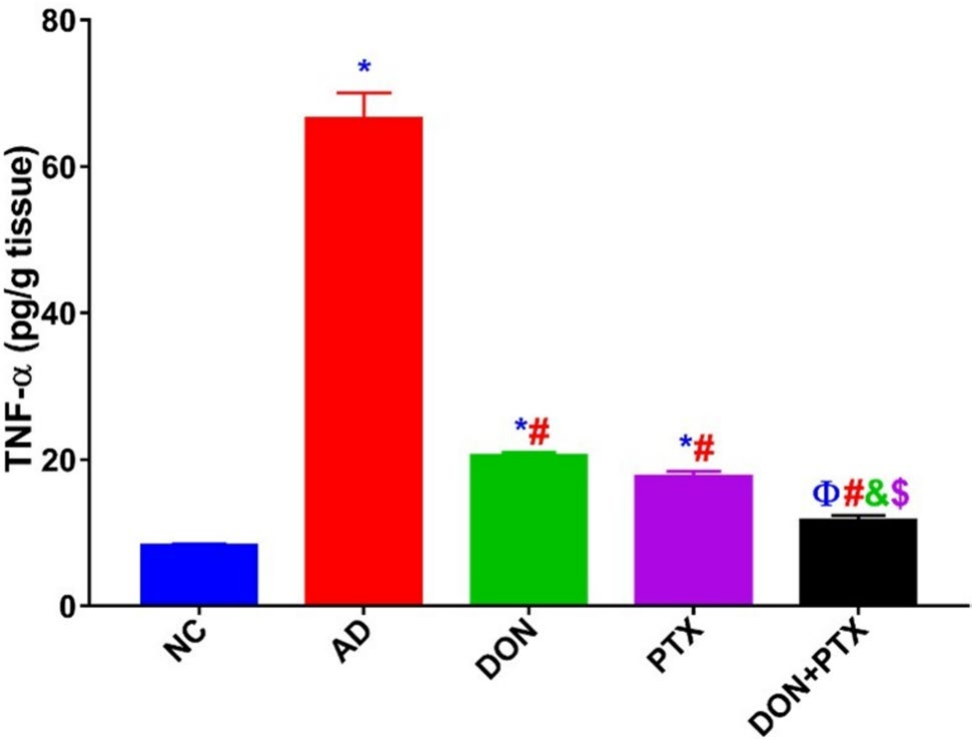
The brain hippocampus TNF-α content in AD rats received DON, PTX, or their combination. Results are expressed as mean ± SD, *n* = 6. *P < 0.001 and ^Ф^*P* < 0.05 compared to the NC group, ^#^*P* < 0.001 compared to the AD group, ^&^*P* < 0.001 compared to the DON group, ^$^*P* < 0.001 compared to the PTX group

AD rats had noticeably increased brain hippocampus TNF-α content (*P* < 0.001) compared to the NC group. The treatment of AD rats with DON, PTX, or their combination significantly decreased the hippocampus TNF-α content compared to the untreated AD rats (*P* < 0.001). Both DON and PTX had comparable results, while their combination significantly decreased (*P* < 0.001) hippocampus TNF-α content compared to individual treatments (Fig. 5).

### Effects of DON and PTX on Apoptotic Biomarkers

**Fig. 6:**
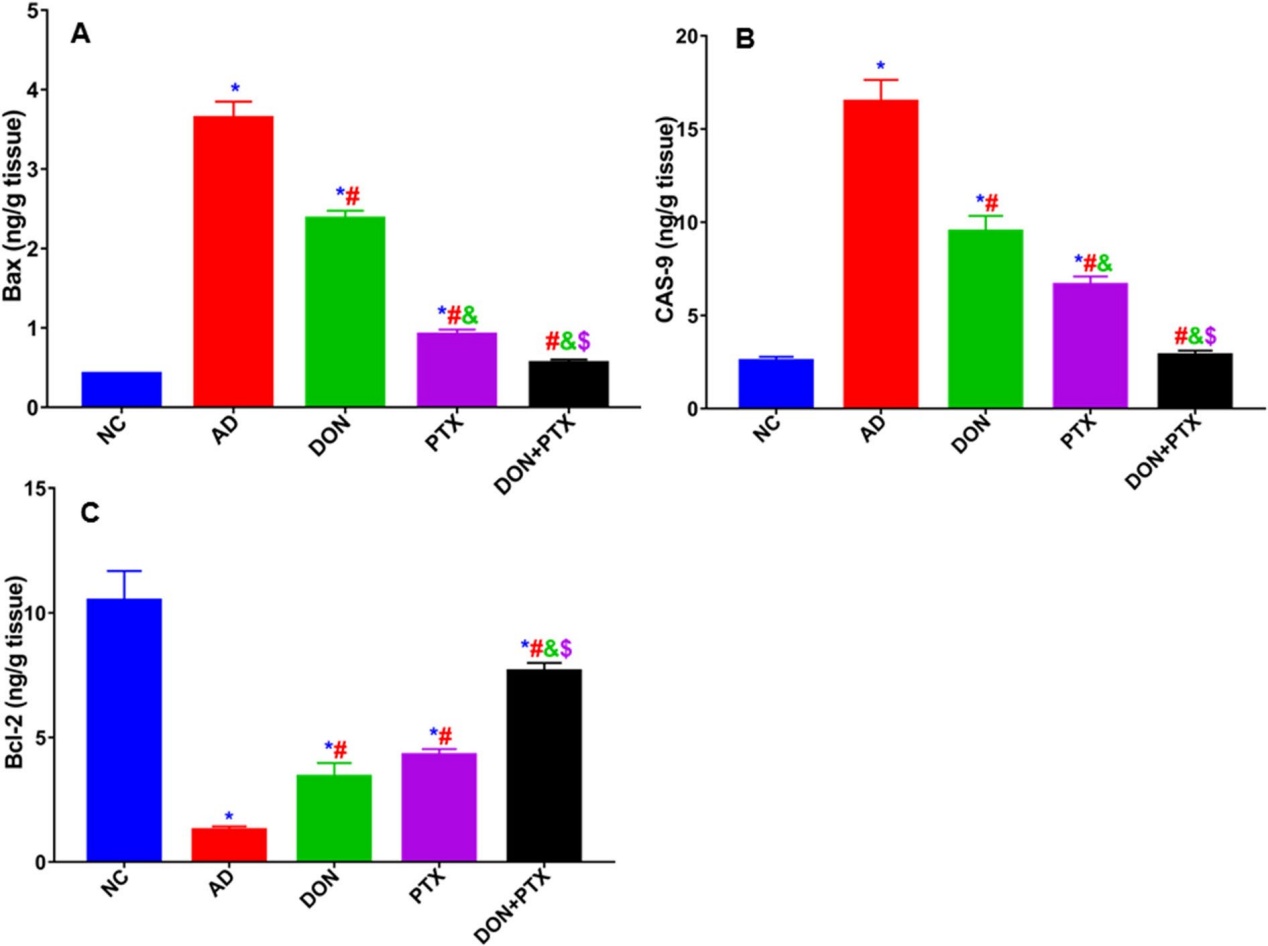
Hippocampus apoptosis biomarkers **A** Bax, **B** CAS-9, and **C** Bcl-2 in rats received CuSO_4_ and treated with DON, PTX, or their combination for 4 weeks. Results are expressed as mean ± SD, *n* = 6. *P < 0.001 compared to the NC group, ^**#**^*P* < 0.001 compared to the AD group, ^**&**^*P* < 0.001 compared to the DON group, ^**$**^*P* < 0.001 compared to the PTX group

AD rats revealed higher hippocampus content of Bax and CAS-9 than rats from the NC group (*P* < 0.001). These were significantly decreased by DON, PTX, or their combination (*P* < 0.001). On the other hand, the hippocampus content of Bcl-2 was lower in CuSO_4_-treated rats than in NC rats (*P* < 0.001). Individual drug treatment as well as their combination increased hippocampus Bcl-2 content compared to the untreated AD group (*P* < 0.001). Combined treatment induced better results than individual treatments (*P* < 0.001), as shown in Fig. 6.

### Effects of DON and PTX on Oxidative Stress Markers

**Fig. 7:**
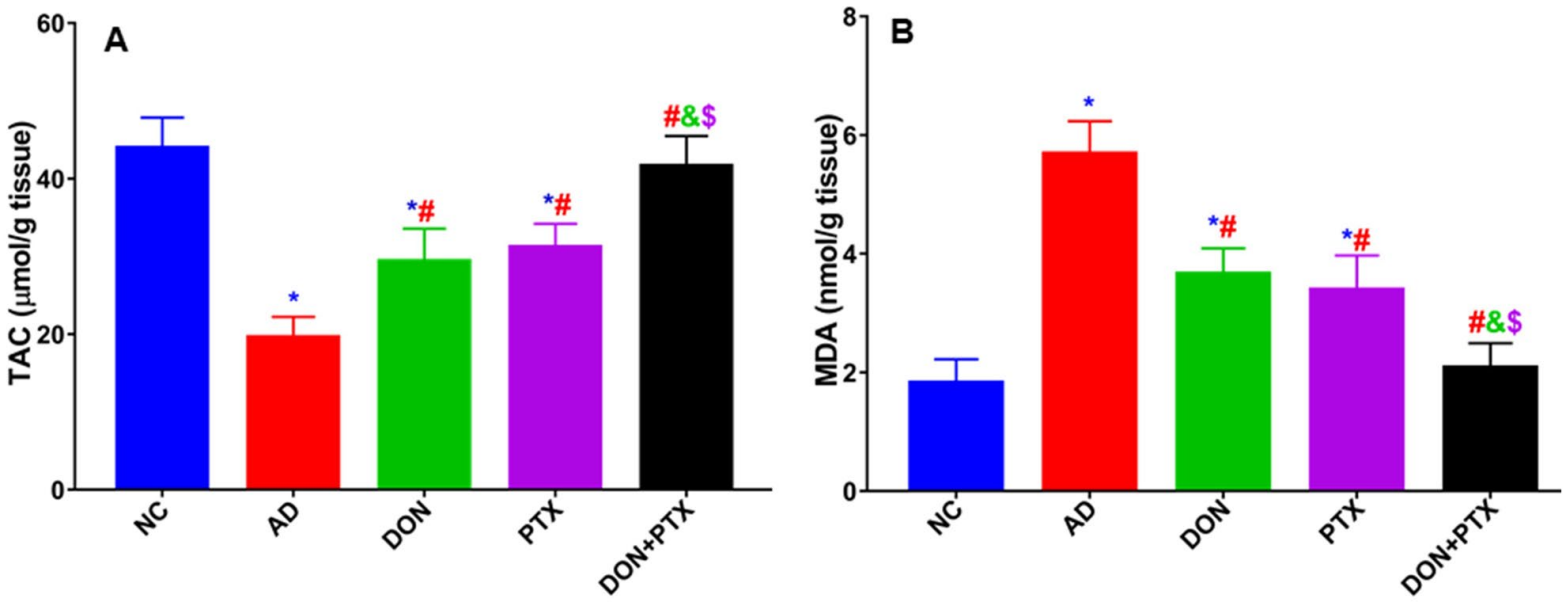
The brain cortical content of oxidative stress markers **A** TAC and **B** MDA in rats received CuSO_4_ and treated with DON, PTX, or their combination. Results are expressed as mean ± SD, *n* = 6. *P < 0.001 compared to the NC group, ^#^*P* < 0.001 compared to the AD group, ^&^*P* < 0.001 compared to the DON group, ^$^*P* < 0.001 compared to the PTX group

Rats received CuSO_4_ (AD rats) demonstrated lower cortical TAC content along with higher MDA content than NC rats (*P* < 0.001). Treatment with PTX, DON, or their combination significantly increased TAC and decreased MDA levels compared to the AD group (*P* < 0.001). Results from groups that received combination treatment were remarkably better than individual treatments (*P* < 0.001), as shown in Fig. 7.

### Hematoxylin and Eosin (H and E) Staining

**Fig. 8:**
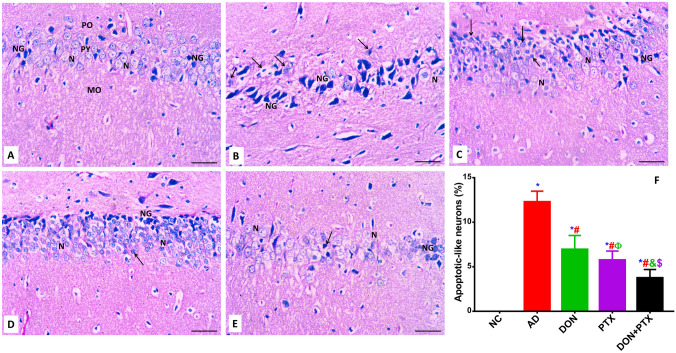
H and E-stained sections of different experimental groups. The CA1 of **A** the NC group showing molecular layer (MO), pyramidal layer (PY), and polymorphic (PO) with bright vesicular nerve cell bodies (N) and darkly stained nuclei of the neuroglial supporting cells (NG). **B** AD group showed nerve cells with darkly stained nuclei (arrow) with increased number of neuroglial cells (NG). Some neurons were noticed with bright nuclei (N). **C** DON-treated group showed increased neuronal cell mass (N) with their supporting neuroglial cells (NG). Some neurons were also seen with darkly stained nuclei (arrow). **D** PTX-treated rats showed a better improvement in the nerve cell population (N) with their supporting cell (NG). Few neurons were noticed with darkly stained (arrow). **E** The combination of both DON with PTX produced a picture almost close to normal histological structure; neurons with bright vesicular nuclei (N) with their supporting cells (NG) and few cells with darkly stained nuclei (arrow). H and E, scale bar 50 µm, 400 × . **F** The percent of neuron cells with histological features of apoptosis (apoptotic-like neurons) in different experimental groups. Results are expressed as mean ± SD, *n* = 6 in triplicates. *P < 0.001 compared to the NC group, ^#^*P* < 0.001 compared to the AD group, ^&^*P* < 0.001 and ^Ф^*P* < 0.05 compared to the DON group, ^$^*P* < 0.001 compared to the PTX group

The histopathological features of H and E-stained hippocampus from all groups were illustrated in Fig. 8A–F. The proper hippocampus contains 4 regions of Cornu Ammonis (CA) represented as CA1-4. The CA1 region is the first region in the hippocampal conduit that produces a significant yield pathway to the entorhinal *subiculum* and *entorhinal cortex*. The CA1 neurons of the hippocampus of the NC rats stained with H and E (Fig. 8A) contain three layers: molecular, pyramidal, and polymorphic layers. The pyramidal nerve cells are tightly crowded with large rounded bright vesicular nuclei and extensive cytoplasmic processes directed toward the molecular layer. The neuropil was packed with neuroglial cells, unmyelinated axons, and dendrites. The nuclei of the neuroglia were detected. In CuSO_4_-induced AD rats (Fig. 8B), the hippocampal CA1 region showed intensely stained acidophilic cytoplasm of some pyramidal cells. The nuclei were rather poorly demarcated or fragmented, with a substantial decline in the pyramidal nerve cell mass. Some pyramidal nerve cells showed the vesicular nuclei. Numerous dark neuroglial cells were also noticed near nerve cell bodies. The administration of DON (Fig. 8C) prevented such neuronal cell loss in the CA1 area of the hippocampus. The population of the pyramidal nerve cells of the CA1 area showed a significant rise in the density with bright rounded vesicular nuclei with prominent nucleoli. Treatment of rats with PTX (Fig. 8D) produced a marked improvement in the neuronal cell mass where neurons of the CA1 regions showed bright vesicular nuclei with prominent nucleoli. The neuropil showed normal structure. The combination of both DON with PTX (Fig. 8E) produced significant effects on the nerve cell density and neuropil with a picture almost close to normal histological architecture.

### The Deposition of Amyloid Plaques in Different Experimental Groups

**Fig. 9:**
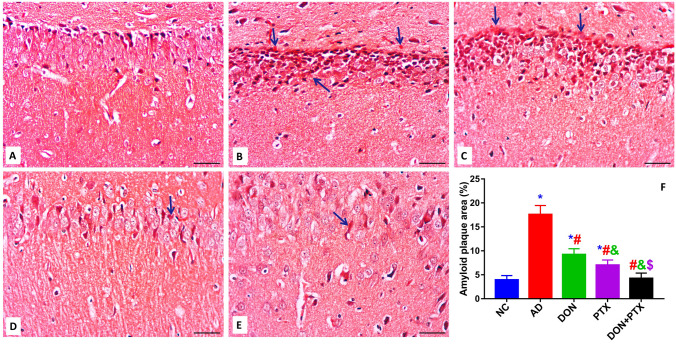
Congo red paraffin-stained hippocampus sections from **A** normal rats, **B** control AD rats, **C** DON-treated rats, **D** PTX-treated rats, and **E** DON + PTX-treated rats. **F** Amyloid deposits revealed by Congo red stain were measured using the ImageJ software. Arrows denote a positive reaction (Congo red stain, scale bar 50 µm, 400 × .). Results are expressed as mean ± SD, *n* = 6 in triplicates. *P < 0.001 compared to the NC group, ^#^*P* < 0.001 compared to the AD group, ^&^*P* < 0.001 compared to the DON group, ^$^*P* < 0.001 compared to the PTX group

Congo red stain was employed to evaluate the deposition of amyloid plaques in the hippocampal tissue. The hippocampal CA1 nerve cells in the NC group (Fig. 9A) were organized with distinctive cell boundaries and clear bright vesicular nuclei. Noticeable nucleoli were also noted. No accumulation of Congo red was seen in normal animals. However, the CA1 neurons in CuSO_4_-treated rats (Fig. 9B) showed an asymmetric architecture and appeared emaciated and bounded by Congo red stains which were prominent in the polymorphic and molecular layers. In contrast, the hippocampus of DON, PTX, and DON + PTX groups (Fig. 9C–E) were presented with few amyloid deposits compared to the hippocampus AD group. The definitely stained deposits were measured with image analysis software to estimate the amount of amyloid deposits (Fig. 9F).

### The Expression of Neurofilaments

**Fig. 10:**
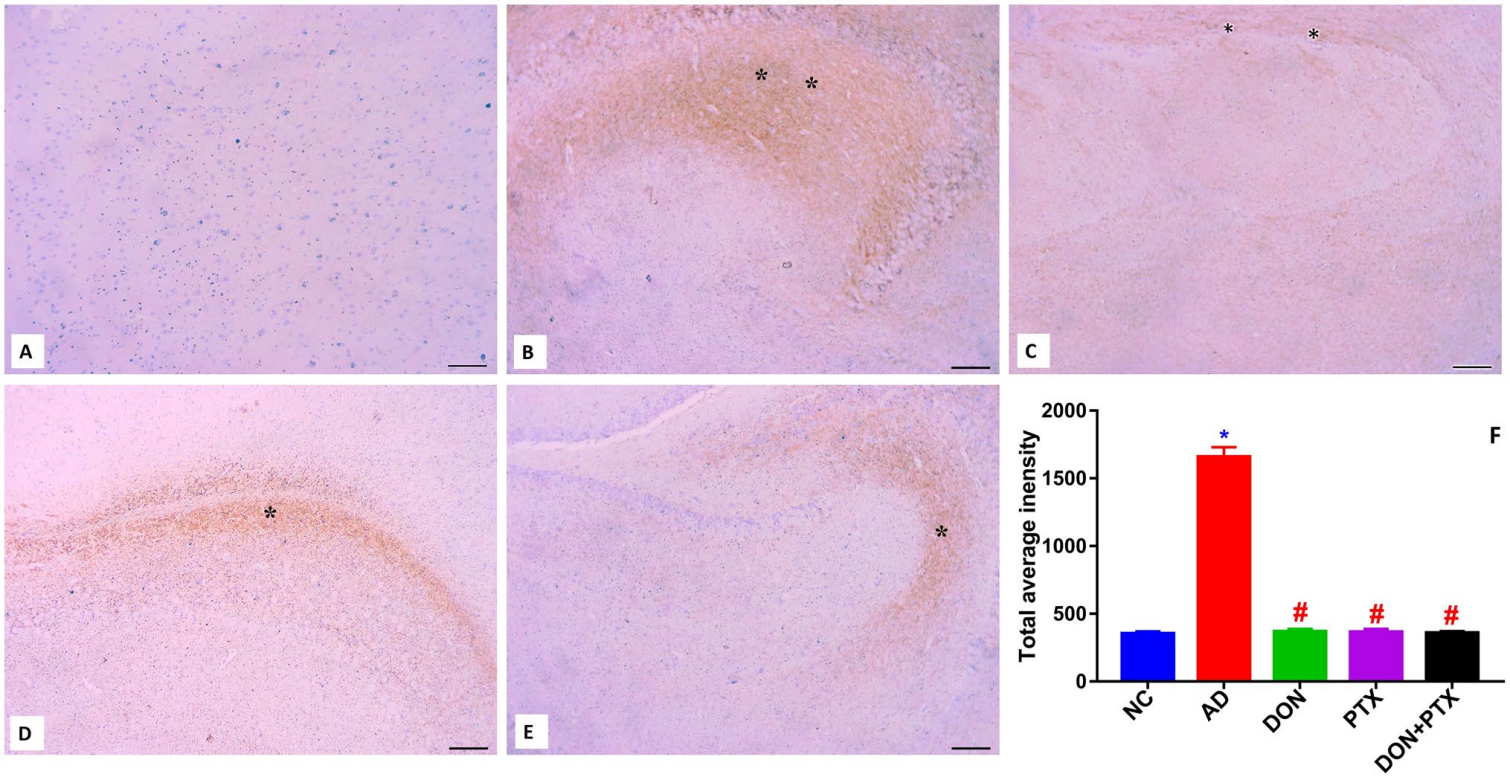
The expression of neurofilaments in the CA1 region of the hippocampus from **A** NC rats, **B** control AD rats, **C** DON-treated rats, **D** PTX-treated rats, and **E** DON + PTX-treated rats. **F** The expression of neurofilaments was measured using the ImageJ software (results were expressed as mean ± SD, *n* = 6 in triplicates, *P < 0.001 compared to the NC group, ^#^*P* < 0.001 compared to the AD group). Asterisks (*) denote positive immunological reaction (neurofibrillary tangles immunostaining, scale bar 30 µm, 100 ×)

Immunohistochemical staining of the CA1 region of the hippocampus of the NC group (Fig. 10A) showed normal pyramidal cells with bright nuclei. The neuropil did not show any pathological alterations. The consumption of CuSO_4_ in drinking water (Fig. 10B) created a marked reduction in the pyramidal cell population and a strong positive reaction to neurofilament compared to control littermates indicating Tau hyperphosphorylation. Treatment with DON (Fig. 10C) produced a marked increase in the pyramidal cell density and reduced the expression of neurofilament compared to control AD rats. Most of the nerve cells exhibit bright nuclei. Treatment with PTX either alone (Fig. 10D) or combined with DON (Fig. 10E) also improved the pyramidal cell viability and suppressed the neurofilament formation compared to control AD rats. The total average intensity of immuno-stained areas was calculated (Fig. 10F).

## Discussion

Inflammatory responses and apoptosis are major factors of AD pathology and its progression. These pathways have attracted many researchers interested in screening and investigating therapies for AD (Wang et al. 2017). As an example of TNF-α level in the cerebrospinal fluid of AD patients is higher than in cognitively normal subjects (Tarkowski et al. 2003; Brosseron et al. 2014). Moreover, TNF-α in brain tissues of AD human and AD animal models was found to be colocalized with amyloid plaques (Kalovyrna et al. 2020). The current study proved that the inhibition of TNF-α using PTX greatly modulated AD pathology and delayed its progression in the AD rat model.

In this study, CuSO_4_ was used to induce AD model in rats, which demonstrated multiple changes in the brain like those involved in AD pathogenesis. Copper (Cu), an essential trace element, is a component of cuproproteins, which are required for a variety of physiological activities including energy production, free radical scavenging, connective tissue formation, iron mobilization, and neurotransmission. Humans are frequently exposed to Cu^2+^ from a variety of sources including drinking water, agrochemicals, and Cu^2+^-containing intrauterine devices (Arowoogun et al. 2021). During Cu^2+^ overload, free Cu^2+^ level may increase which is harmful and will induce an imbalance in cerebral Cu^2+^ homeostasis leading to the development of AD and other neurodegenerative disorders (Singh et al. 2013). Ceruloplasmin (Cp) restoration in the brain of AD mouse could attenuate hippocampus cell damage, indicating that Cp has a neuroprotective function (Zhao et al. 2018). Although the majority of Cu^2+^ in the plasma is stable when coupled to Cp, some of it is unstable when bound to other molecules like albumin and globulin. It was reported that the level of non-ceruloplasmin-bound Cu (non-Cp-Cu) was markedly increased in AD and mild cognitive impairment (MCI) (Squitti et al. 2011), and such increase may predict the progression of MCI to AD. Non-Cp-Cu levels in turn is usually increased in the early stages of MCI (Liu et al. 2022).

The elevated Cu^2+^ content in the brain tissues of patients with cognitive impairments was found to be associated with chronic inflammation as well as declined antioxidant status, all of which are major factors contributing to the pathological progression of AD (Arowoogun et al. 2021). The current study illustrated increased inflammation and lipid peroxidation along with decreased antioxidant capacity in the control AD group. These results are in agreement with previous studies (Ali et al. 2021; Chen et al. 2021; Tayanloo-Beik et al. 2022). Cu^2+^ exposure was previously reported to increase inflammatory responses and inhibits Aβ clearance in the brain (Giacconi et al. 2019; Yin et al. 2019). The generation of ROS is a crucial contributor to β-amyloid toxicity toward neurons. In the presence of biological reducing agents, Cu^2+^ in combination with β-amyloid fibrils form hydrogen peroxide (Parthasarathy et al. 2014), while the removal of Cu^2+^ from β-amyloid inhibits amyloid aggregation in vitro accelerates β-amyloid breakdown and reduces the production of H_2_O_2_ (Bagheri et al. 2017).

Furthermore, Cu^2+^ was found to build up in the amyloid plaque of AD individuals which is considered as a hallmark in the development of AD. In this context, the current CuSO_4_-induced AD rat model showed a significant accumulation of amyloid plaques in the hippocampal tissue as revealed in Congo red-stained hippocampus. The amyloid deposits typically exhibit a β-sheet secondary structure that aggregates leading to the formation of fibrils and plaques. Congo red dye binds to the β-pleated sheet structure of amyloid fibrils by hydrogen bonds staining compact amyloid protein aggregates (Wilcock et al. 2006). Additionally, the interaction of Cu^2+^ with Aβ is related to the formation of oligomers capable of entering cells. Therefore, Cu^2+^ toxicity in AD was thought to be mediated by Aβ-bound Cu^2+^ inhibiting cytochrome-c oxidase and their potential to induce Tau protein phosphorylation and aggregation via cysteine residues (Ayton et al. 2013; Hayne et al. 2014). These Tau aggregates are seen in the early stages of AD and could be useful biomarkers for early diagnosis and treatment of the disease (Rajasekhar and Govindaraju 2018). Our results showed an increased formation of p-tau and neurofilaments in CuSO_4_-induced AD rat model and in agreement with previous studies (Kitazawa et al. 2009; Voss et al. 2014).

Clusterin (CLU), commonly known as apolipoprotein J (ApoJ), was also increased in the brain of CuSO_4_-induced AD rat model. Similar increase of brain CLU was previously reported (Miners et al. 2017; Jackson et al. 2019). CLU was reported to be linked to increased AD risk and the severity of cerebral amyloid angiopathy (Bettens et al. 2013). It was also found to be physically linked with Aβ-rich extracellular plaques in the brains of AD patients and roughly reflects the regional distribution of Aβ (Wilson and Zoubeidi 2017).

During Aβ formation, BACE1 catalyzes the rate-limiting initial cleavage at the site of amyloid precursor protein (APP), which is followed by successive intra-membrane processing at numerous locations by y-secretase (Jonsson et al. 2012; Vassar et al. 2014). BACE1 protein expression was also increased in the brain tissues of CuSO_4_-induced AD rat model. BACE1 is not just a biomarker for AD, but it also causes cognitive impairment (Yue et al. 2020).

AD is characterized by a substantial loss of cholinergic innervation. The concentration of ACh is markedly reduced in the hippocampus and the cerebral cortex of AD patients (Ullrich et al. 2010). The link between Aβ accumulation and cholinergic neurotransmitter system is bi-directional, where (i) AChE can stimulate the formation of Aβ and its incorporation into the growing Aβ-fibrils and (ii) Aβ can impair the release of ACh by interacting with choline transporter and inhibiting ACh biosynthesis (Pakaski and Kalman 2008). Oxidative stress, in addition, can increase the activity of AChE leading to a decline in cholinergic activity. The current study in accordance revealed increased cortical AChE activity along with cortical ACh depletion in CuSO_4_-induced AD rat model. Since the major inputs of ACh to the hippocampus are provided by the basal forebrain cholinergic populations which are selectively impacted in AD manifesting the impairment of hippocampus-dependent memory. Therefore, the hippocampus-dependent learning is modulated by ACh in the basal forebrain (Haam and Yakel 2017). Accordingly, the learning ability and spatial cognition of CuSO_4_-induced AD rat model (shown in Y-maze test) are affected as this cholinergic system is crucial for numerous physiological processes such as attention, memory, and learning (Melo et al. 2003).

Apoptosis is another molecular change which is involved in neurodegenerative diseases and AD pathogenesis. Progressively higher levels of total CAS-9 and other pro-apoptotic proteins Bax, CAS-3, and CAS-8 were found in platelet-rich plasma from patients with amnesic mild cognitive impairment (a loss of cognitive function that can lead to AD diagnosis) and AD compared to subjects without cognitive deficits (Zhao et al. 2016). Results from the current investigation showed increased expression of both CAS-9 and Bax, along with decreased expression of Bcl-2 in the brain hippocampus of CuSO_4_-induced AD rat model. Consequently, increased Bax/Bcl2 ratio in CuSO_4_-induced AD rat model favoring apoptosis that was also confirmed by increased number of neuron cells with histological features of apoptosis (apoptotic-like neurons) seen in H and E-stained hippocampus sections. The condensation of chromatin and cytoplasmic content of affected neurons makes them appear smaller than the surrounding cells with a small darkly stained pyknotic nucleus with acidophilic cytoplasm. These results are in accordance with previous studies (Zhao et al. 2016; Liu et al. 2018; Su et al. 2020).

The activation of caspases was reported to cause an early synaptic impairment in the AD mouse model (D’Amelio et al. 2011). In Tau transgenic mice, active caspases also cleave Tau to start tangle formation (de Calignon et al. 2010). As a result, maintaining healthy mitochondria is critical not only for preventing energy failure in the AD brain, but also for inhibiting caspase activation, such as CAS-9 and CAS-3, and therefore preventing synaptic dysfunction, tangle formation, and neurodegeneration (Fossati et al. 2016). In other words, increased apoptosis reported in this CuSO_4_-induced AD rat model may explain the increased formation/deposition of amyloid plaques (Congo red staining, Fig. 9) and the neuron-specific axonal cytoskeletal protein (neurofilaments reported by immunostaining, Fig. 10).

Treatment of the CuSO_4_-induced AD rats with either PTX or DON revealed significantly reduced TNF-α improved cholinergic activity (increased cortical ACh content along with decreased AChE activity). Both drugs decreased apoptotic activity (decreased hippocampal apoptotic-like neurons, Bax and CAS-9, while increased anti-apoptotic Bcl-2). Consequently, AD markers, namely hippocampus BACE1, p-tau, and CLU, and the hippocampal expression of Aβ and neurofibrillary tangles were also decreased leading to improved spatial cognition ability.

Although DON, a selective AChE inhibitor, is commonly used to treat mild, moderate, and severe degrees of AD, as well as vascular dementia and dementia linked to Parkinson’s disease (Jiang et al. 2019), the results of the current investigation showed that PTX revealed better outcomes than DON in modulating AChE activity, Bax, CAS-9, BACE1, CLU, and p-tau. The modulation of AD pathogenesis was remarkable using PTX and DON in combination. The better outcomes of this combination therapy can encourage the recommendation of their combined use for AD.

## Conclusion

The use of anti-inflammatory drug, PTX, inhibited hippocampal TNF-α and modulated hippocampal apoptosis, amyloid plaque deposition, Tau aggregation, and cortical cholinergic activity and therefore resulted in improved cognition in the CuSO_4_-induced AD rat model. The use of PTX in combination with DON has offered great potential toward decreasing AD pathogenesis in this rat model. However, more studies are still required to further confirm and prove the long-term efficacy of such combination and their effect on other AD models.

## Data Availability

Data are available to the corresponding author upon reasonable request.
